# Real-time monitoring of the dynamics and interactions of bacteria and the early-stage formation of biofilms

**DOI:** 10.1038/s41598-022-22669-0

**Published:** 2022-10-28

**Authors:** Francesco Giorgi, Judith M. Curran, Eann A. Patterson

**Affiliations:** School of Engineering, University of Liverpool, Brownlow Hill, Liverpool, L69 3BX UK

**Keywords:** Bacteria, Biofilms

## Abstract

Bacterial biofilms are complex colonies of bacteria adhered to a static surface and/or one to another. Bacterial biofilms exhibit high resistance to antimicrobial agents and can cause life-threatening nosocomial infections. Despite the effort of the scientific community investigating the formation and growth of bacterial biofilms, the preliminary interaction of bacteria with a surface and the subsequent early-stage formation of biofilms is still unclear. In this study, we present real-time, label-free monitoring of the interaction of *Escherichia coli* and *Pseudomonas aeruginosa* bacteria with untreated glass control surfaces and surfaces treated with benzalkonium chloride, a chemical compound known for its antimicrobial properties. The proof of principle investigation has been performed in a standard inverted optical microscope exploiting the optical phenomenon of caustics as a tool for monitoring bacterial diffusion and early adhesion and associated viability. The enhanced resolving power of the optical set-up allowed the monitoring and characterization of the dynamics of the bacteria, which provided evidence for the relationship between bacterial adhesion dynamics and viability, as well as the ability to form a biofilm. Viable bacteria adhered to the surface exhibited noticeable sliding or rotary dynamics while bacteria killed by surface contact remained static once adhered to the surface. This difference in dynamics allowed the early detection of biofilm formation and offers the potential to quantify the efficiency of antimicrobial surfaces and coatings.

## Introduction

Biofilms are complex dynamic microbial communities colonising and growing on surfaces. The transition from the individual planktonic state (single bacteria cell floating in solution) to a biofilm enhances the tolerability of bacteria to antibiotics and their growth even in unfavorable conditions^1^. Within the biofilm, bacteria are enclosed in a self-produced extracellular matrix, consisting of extracellular polymeric substances (EPS) that, along with carbohydrate-binding proteins, adhesive structures such as pili and flagella, and extracellular substances, act as a stabilizing scaffold for the three-dimensional structure of the biofilm. This structure provides bacteria with the appropriate amount of nutrients needed to favour their growth and reproduction, enhancing cell-to-cell interactions, DNA exchange, and protecting the biofilm components from drying, predation, and other external damaging agents^2^.

**Figure 1:**
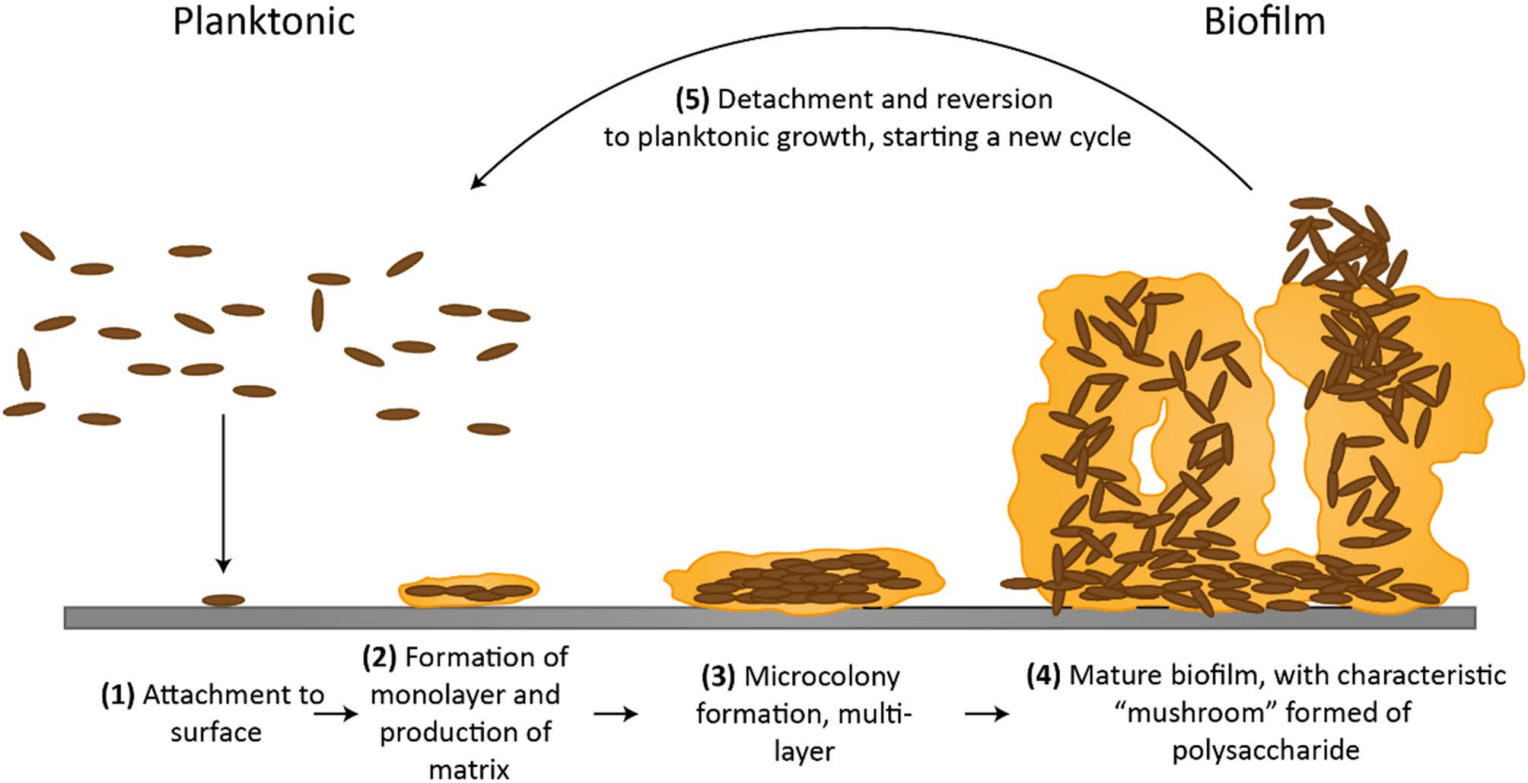
Schematic representation of the five stages of biofilm formation^8^.

The mechanisms of bacterial adhesion and biofilm formation are governed by several physical, chemical and biological factors. The first phase of biofilm formation concerns the individual attachment of a single bacterium to a surface. Individual bacterial cells are transported to the surface either by physical forces or by an intrinsic locomotion ability. Motile bacteria use structures, such as flagella, to approach the surface, guided by chemotactic, aerotactic, or phototactic responses. Motility promotes both initial interaction with the surface and movement along it. On the other hand, non-motile cells, are delivered to the surface by diffusion and sedimentation processes or by the flow of the fluid in which they are suspended^3^. Once a bacterium has approached the surface, the initial attachment is regulated by: attractive and repulsive forces, mainly Van der Waals and electrostatic interactions; the properties of the surface such as texture, roughness, and hydrophobicity; and, the properties of the solution such as pH and temperature^4^. However, attached bacteria can detach from the surface and rejoin the planktonic state in a process called reversible adhesion, as a result of hydrodynamic forces, repulsive forces, or in response to nutrient availability^5^. If the environmental conditions are favourable, a bacterium attaches permanently to the surface and starts to secrete EPS, establishing a permanent bond with the surface (known as irreversible adhesion) and favouring the attachment of additional bacteria^6^. Irreversibly attached bacteria continue to secrete EPS, forming a micro-niche favourable to their survival, their proliferation and their cohesion. The presence of two-dimensional microcolonies of interacting bacteria attached to a surface represents the early phase of the formation process of biofilms^7^. The bacterial biofilm develops from a two-dimensional monolayer of irreversibly attached bacteria to a three-dimensional multilayer colony which continues to grow, projecting into the surrounding medium for hundreds of microns. This biofilm structure acts as a primitive circulatory system, connecting the individual cells and allowing nutrient exchange and waste removal. In this final stage, the biofilm is eventually broken by the pressure caused by the ever-growing number of bacteria or by the action of external forces, such as fluid shear or abrasion. The bacteria are then dispersed again into the surrounding medium, able to colonise and infect a new substrate (Fig. 1)^8^.

Bacterial biofilms account for up to 80% of nosocomial infections in the US, and due to their adaptability and higher resistance to antibiotics, bacterial biofilms can easily form on medical devices and human tissues, leading to chronic and life-threatening infections^9^. Hence, methodologies to prevent biofilm formation or activate the immune system to eradicate these communities are vital for the effective control of biofilm-related diseases.

Several strategies have been investigated previously to prevent bacterial biofilm formation and subsequent infection, with the development of a range of so-called antimicrobial surfaces. The majority of these surfaces are designed to kill bacteria on contact or close proximity by the release of antimicrobial substances (biocidal surfaces)^10^.

Despite significant efforts to characterise and prevent biofilm formation and because of the variety of forces involved, a clear understanding of the preliminary bacteria–surface interaction and the subsequent early-stage biofilm formation is needed to develop effective antimicrobial strategies and to support their translation to in-vivo and real-life scenarios. In this study, we have monitored in real-time the dynamics and surface interactions of *E. coli* and *P. aeruginosa* bacteria exposed to glass surfaces, with or without an antimicrobial coating, without labelling the bacteria. *E. coli* is a Gram-negative, rod-shaped, non-sporulating bacterium considered as a model organism for studies in biological engineering and industrial microbiology. Most *E. coli* strains are harmless to the human body and are found in the intestine of warm-blooded organisms. However, several *E. coli* strains are pathogenic and responsible for infections in various medical devices, such as urethral and intravascular catheters, prosthetic joints, shunts, and grafts^11^. *E. coli* biofilm can be also responsible for skin and soft-tissue infections^12^. *P. aeruginosa* is a Gram-negative, rod-shaped, asporogenous, and monoflagellated bacterium, known for causing serious infections in immuno-compromised patients with cancer and patients suffering from severe burns and cystic fibrosis^13^.

Bacteria imaging is commonly performed using fluorescence microscopy because it enhances the low resolving power and low contrast achievable with common brightfield microscopy. However, given the limitations of fluorescence microscopy, including photobleaching and phototoxicity, and the lack of knowledge about the effect on bacterial functions and processes caused by the exposure to fluorescent dyes, strategies have been developed to perform label-free monitoring of bacterial cells and their initial adhesion to a surface. Amongst other techniques, phase-contrast microscopy is one of the most effective label-free optical techniques in terms of resolution and contrast of the acquired image, allowing for the three-dimensional tracking of a bacterial cell in simple and complex environments^14^. However, phase-contrast microscopy requires a specialized and expensive optical set-up, equipped with a specific condenser with a condenser annulus coupled with objectives characterized by the presence of phase plates responsible for retardation of the diffracted rays^15^. Another optical technique complementary to phase contrast microscopy, and able to produce high contrast images of biological organisms, is differential interference contrast microscopy. The high resolution and contrast are achieved by the usage of two interfering coherent beams. The technique is capable of visualizing human^16^ and bacterial cells^17^; however, it requires a specialized and costly optical set-up, equipped with a series of polarizer and prisms.

In this study, imaging has been performed by generating caustic signatures of bacteria populations, allowing monitoring of them in a common inverted optical microscope with only some simple adjustments and without any requirement for fluorescent labelling. The high resolving power of the optical setup based on caustics allowed the qualitative characterization of the dynamics of *E. coli* and *P. aeruginosa* bacteria and the detection of preliminary bacteria-surface interactions, providing real-time information about bacteria forming a biofilm and proliferating on the target surface.

The aim of the study was to investigate the mechanism of attachment of bacteria to surfaces and to characterise qualitatively the potential relationship between the dynamics exhibited by bacteria when interacting with the surface and their viability or ability to start a biofilm. The results provide evidence of relationships between bacteria dynamics and the formation of a biofilm which has direct implications for the characterization of the efficiency of antimicrobial surfaces as well as for the assessment of the early-stage formation of biofilms. The technology described and employed in this study can be used to generate data in the form of real-time images and thereby facilitate cost-effective label-free studies of the efficiency of antimicrobial surfaces in vitro.

## Material and methods

Bacterial imaging has been performed on non-motile *E. coli* ATCC 10536 strain and on motile *P. aeruginosa* ATCC 15442 strain. Both strains are commonly used for antimicrobial investigations. Overnight cultures of bacteria were diluted to McFarland Standard 0.5 in Luria–Bertani (LB) broth or phosphate-buffered saline solution (1× PBS, pH 7.4) as appropriate to obtain a working concentration of approximately 10^8^ CFU/mL. Bacteria were diluted in pure PBS or in solution of 10% v/v LB in PBS. The amount of LB was limited to a maximum concentration of 10% v/v to limit the growth and ability to proliferate of the bacteria, thus allowing longer monitoring times for the interaction between single bacterium and the target surface and the formation of the biofilm. The low limit of LB also minimised the caustic signatures generated from the nutrients in the medium (for example, see Fig. 1 in the supplementary material). Bacterial dynamics and interactions were monitored in a standard optical inverted microscope (Axio Observer.Z1 m, Carl Zeiss), mounted on antivibration feet (VIBe, Newport) to isolate the sample from the environment, using 60 μl of bacteria solution in a deep cavity (250 ± 10 μm in depth) in microscopy slides. The microscope was equipped with a monochrome camera (AxioCam ICm1, Carl Zeiss) to acquire images and record videos at up to 30 fps and with a stage-top incubation system (Incubator PM S1, Heating Insert P S1, Temp and CO_2_ module S1, Carl Zeiss) to control the temperature and the amount of CO_2_ present during the experiments. The resolving power of the optical setup was increased by making some simple adjustments to the normal set up of the microscope following the procedure described by Patterson and Whelan to generate caustic signatures of the bacteria^18^. The caustic optical signatures facilitated the recognition of the structural morphology of the bacteria and the identification of single bacterium and clusters of bacteria, as well as the dynamics of their interactions. Scanning electron microscopy requires the drying and fixation of the bacteria population which arrests the dynamic interactions, hence, to validate the label-free caustic signatures associated with bacteria in solution and adhered to the surface, fluorescence images of bacteria exposed to control glass surfaces were acquired with the same optical set-up using a fluorescent light source. *E. coli* bacteria were stained with the fluorescent dyes SYTO9 and propidium iodide (PI) from the LIVE/DEAD BacLight kit (Invitrogen), a well-known fluorescence kit used to assess the viability of bacteria in solution. SYTO 9 is a green-fluorescent nucleic acid stain able to permeate the membrane of both live and dead Gram-positive and Gram-negative bacteria, while PI is a red-fluorescent nuclear and chromosome stain which is permeant to bacterial cells with disrupted membrane^19^.

The LIVE/DEAD BacLight kit consists of two components:

- Component A: SYTO 9 dye, 1.67 mM/PI dye, 1.67 mM 300 µL solution in DMSO
- Component B: SYTO 9 dye, 1.67 mM/PI dye, 18.3 mM 300 µL solution in DMSO

As both components of the kit consist of SYTO 9 and PI dyes at the same time, we decided to use a different fluorochrome, the trypan blue, to stain the bacteria population exposed to surfaces coated with benzalkonium chloride (BKC). The trypan blue selectively stains only bacterial cells with disrupted membranes, which allowed dead cells to be identified.

The efficiency BKC as an antimicrobial agent and its effect on the bacterial-surface interaction was tested by spreading a 0.1% solution of BKC in DI water on some glass surfaces. The surfaces treated with BKC were left for 2 h, to allow for the complete evaporation of the BKC solution and for the deposition of the BKC film, before solutions of bacteria were exposed to them.

The dynamics of the bacteria and their interactions with the surfaces were evaluated over a time period of 20 s using the ImageJ plugin Trackmate, which provided a way to automatically segment spots or roughly spherical objects from a 2D or 3D image and track them over time^20^.

## Results and discussion

### Optical imaging of *E. coli* bacteria

**Figure 2:**
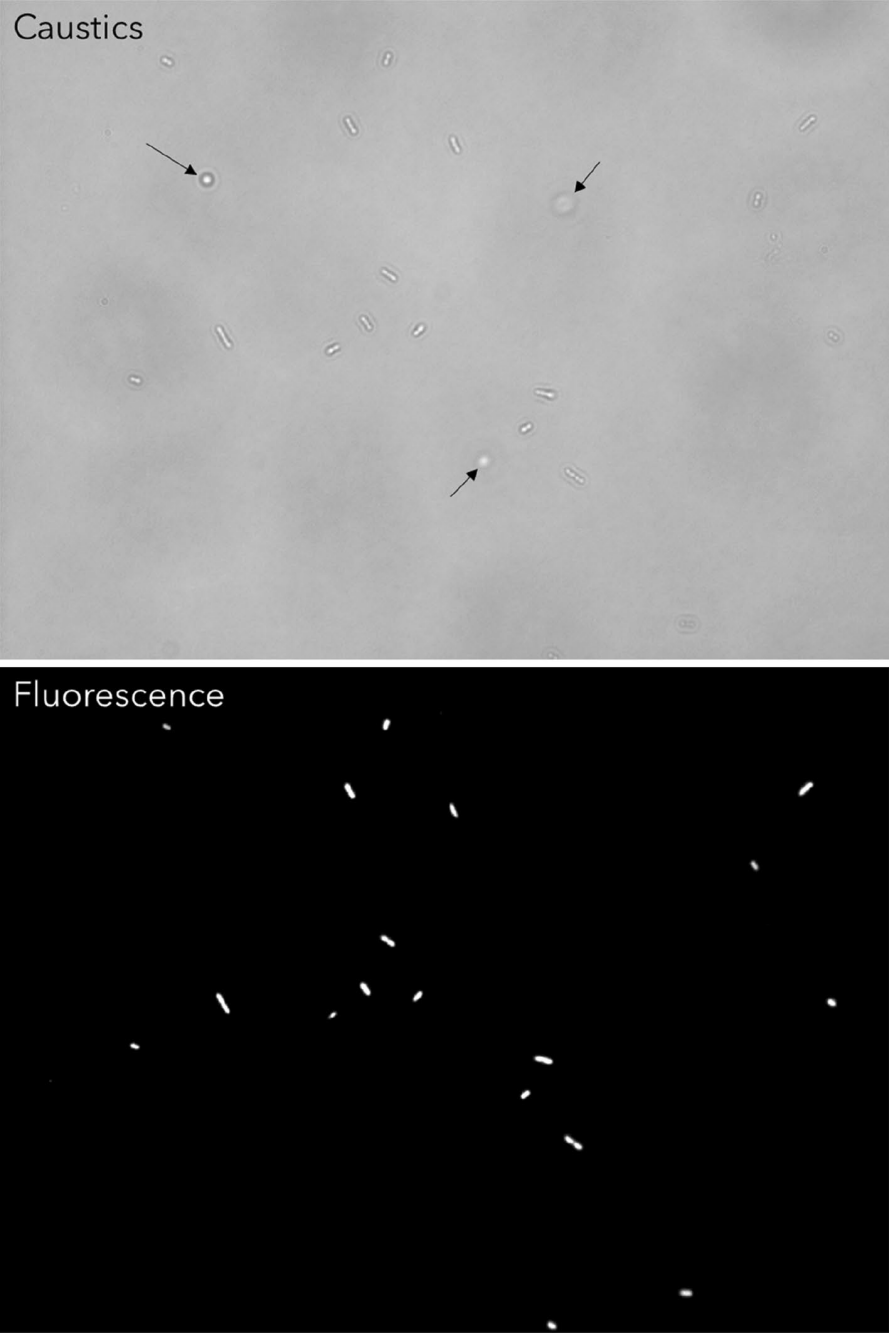
Comparison between the same *E. coli* bacteria population imaged with an inverted optical microscope in caustics mode (top) and fluorescence mode (bottom). *E. coli* bacteria were stained with the fluorescent dyes SYTO9 and propidium iodide (PI) from the LIVE/DEAD BacLight kit. There are no significant differences between the two imaging techniques. The three bacteria highlighted by the black arrows in the caustics image are not at the surface and have been captured when aligned perpendicularly to the surface during their random diffusion, resulting in an almost spherical optical signature.

The imaging capability of the optical setup used in this study to visualize bacteria was validated against fluorescence microscopy by capturing consecutives images of the same region of the sample by switching from the caustic mode to the fluorescence mode which involved using a different light source. Figure 2 shows that there is no noticeable difference between the images captured in the caustic and fluorescence modes, confirming the ability of the caustic technique to generate optical signatures for all of the bacteria present in the field of view, enabling the real-time monitoring and characterisation of the bacteria dynamics and interaction without any requirement for fluorescent labelling. Moreover, the caustic mode allows a better definition of the macroscale structure of the bacteria to be imaged.

**Figure 3:**
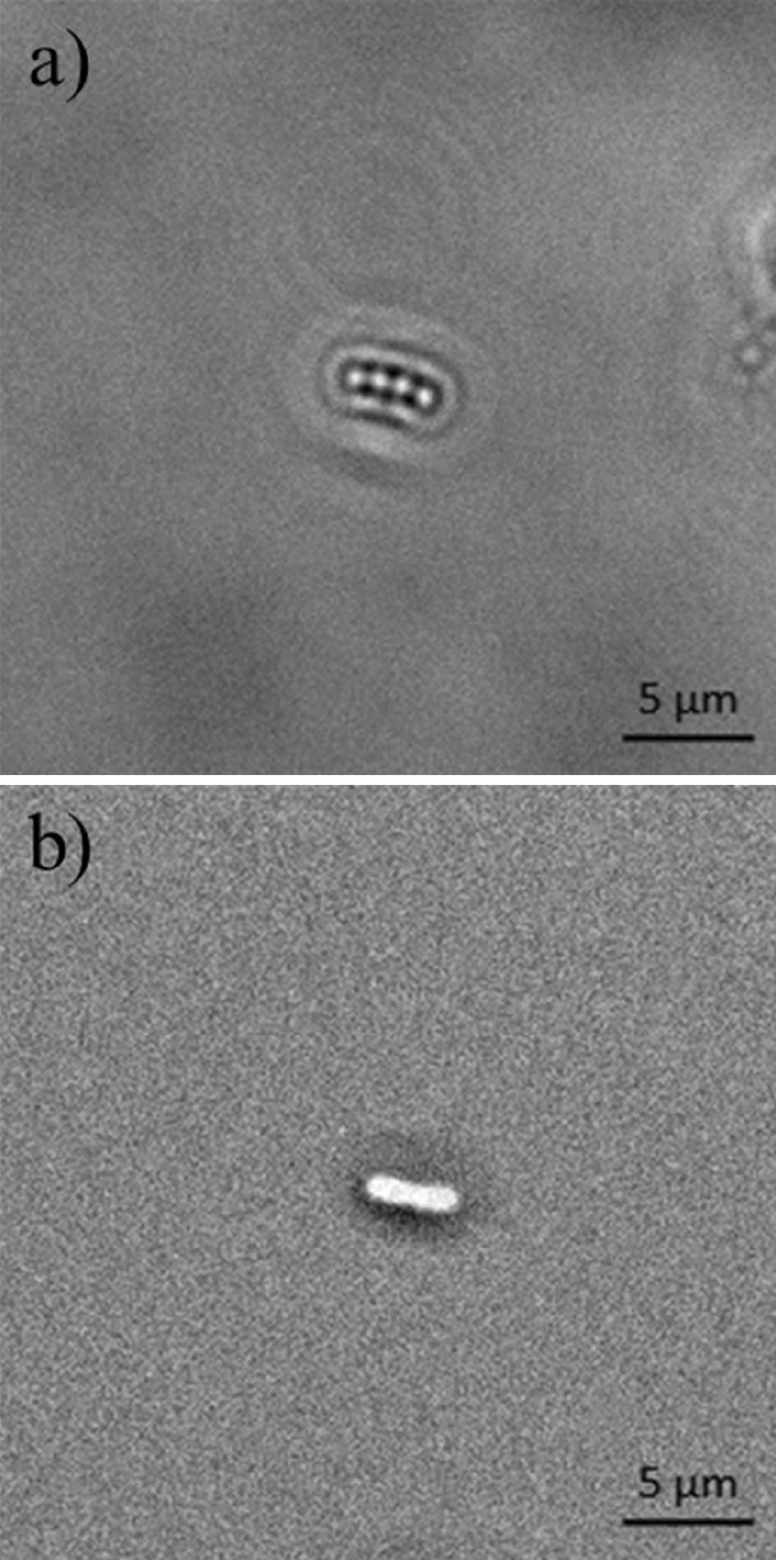
Photograph of the optical signature generated by an *E. coli* bacterium taken with the optical inverted microscope used in this work set-up for: (**a**) caustics mode; (**b**) brightfield mode.

Figure 3 shows a comparison between the typical optical signature of an *E. coli* bacteria adhered to the surface generated using the caustic mode and the brightfield mode. The caustic mode allows for the recognition of the main structural components of the bacteria, namely the membrane, the nucleoids, and the Z rings between them which serves as a scaffold to recruit the FtsZ division proteins, and possibly induces forces that constrict the cell^21^.

### *Escerichia coli*: surface interaction

The interaction of *E. coli* bacteria with surfaces was investigated by characterising the dynamics of bacteria in contact with the surface. Standard glass controls and antimicrobial BKC surfaces were used to investigate the range of bacteria-surface interactions. Standard glass can be assumed to be a positive control which allows bacterial adhesion, whilst BKC was incorporated as a negative control which is accepted as antimicrobial and kills adherent bacteria. Once a bacterium approaches the glass surface, the preliminary interaction is mainly regulated by electrostatic and hydrodynamic interactions. In our experimental scenario, both the bacteria and glass carry a negative surface charge. The outer membrane of *E. coli* is negatively charged due to the dissociation of carboxyl and phosphate groups in the peptidoglycan and lipopolysaccharides of the cell walls^22^, while glass is known to acquire a negative charge in contact with aqueous solutions, mainly because of the dissociation of the silanol groups^23^:

$$\mathrm{SiOH}\to {\mathrm{SIO}}^{-}+{\mathrm{H}}^{+}.$$

**Figure 4:**
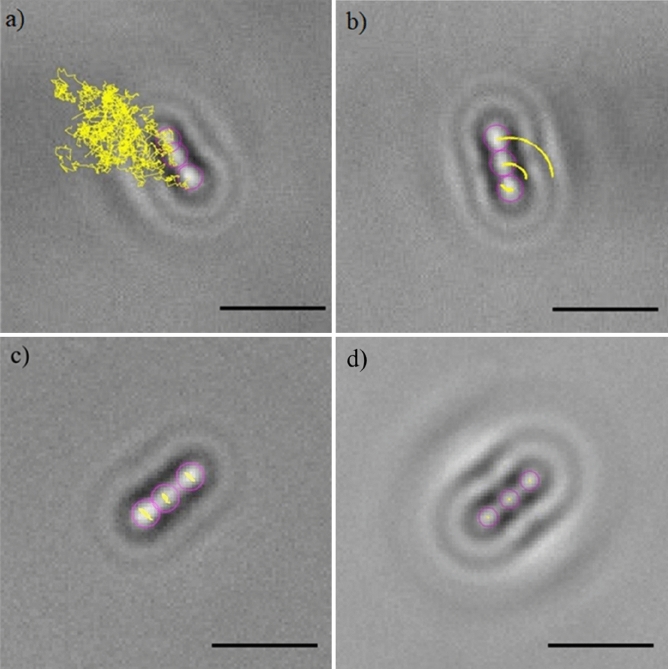
Tracks (yellow lines) of the sections (purple circles) of four *E. coli* bacteria experiencing: (**a**) random diffusion above the surface; (**b**) rotary attachment; (**c**) lateral attachment; (**d**) static attachment. The dynamics of the four bacteria was monitored for approximately 20 s. The length of the scale bars is 5 μm.

**Figure 5:**
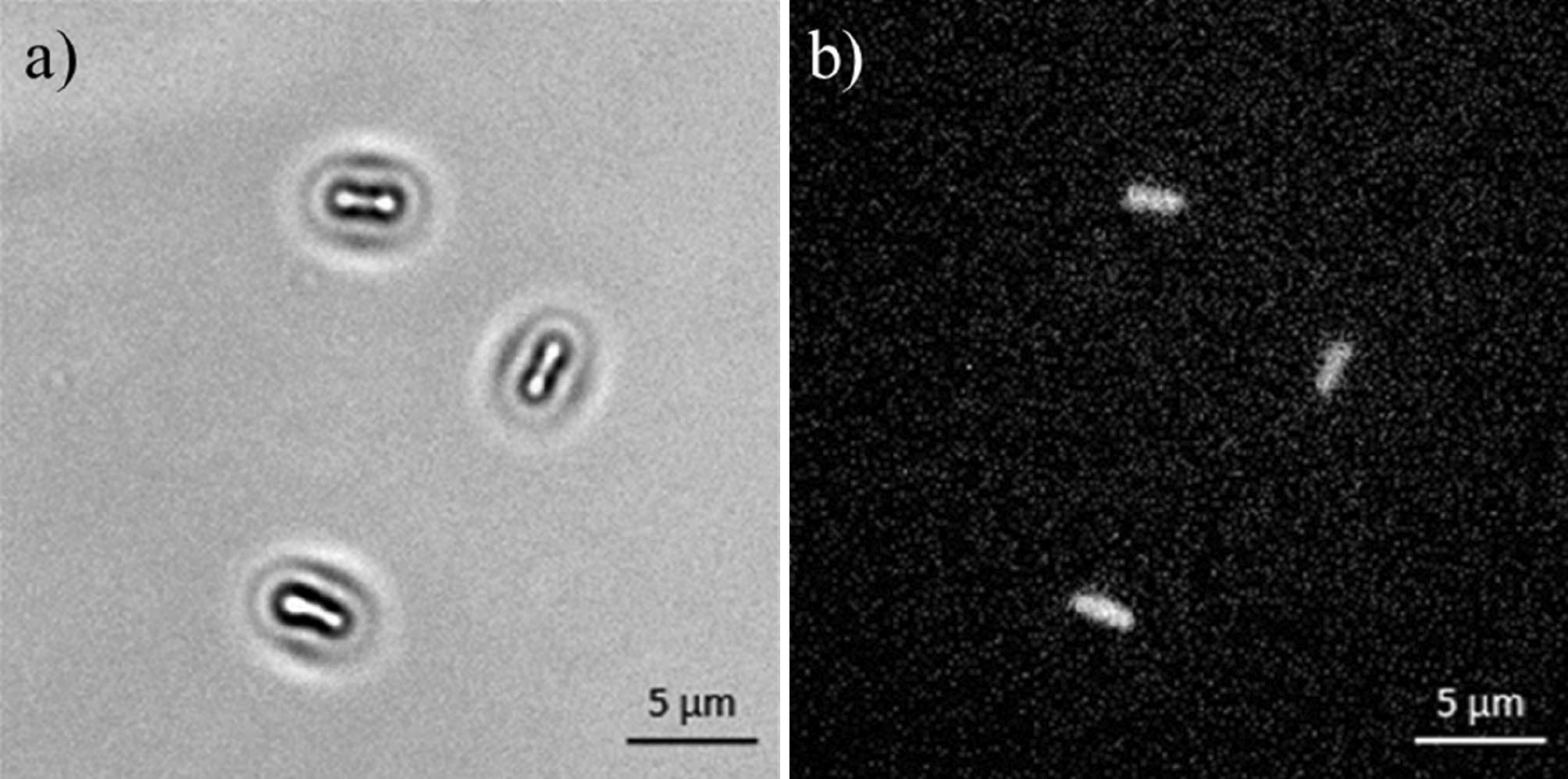
*E. coli* bacteria stained with trypan blue and statically attached to a BKC surface imaged with an inverted optical microscope in caustics mode (**a**) and fluorescence mode (**b**).

**Figure 6:**
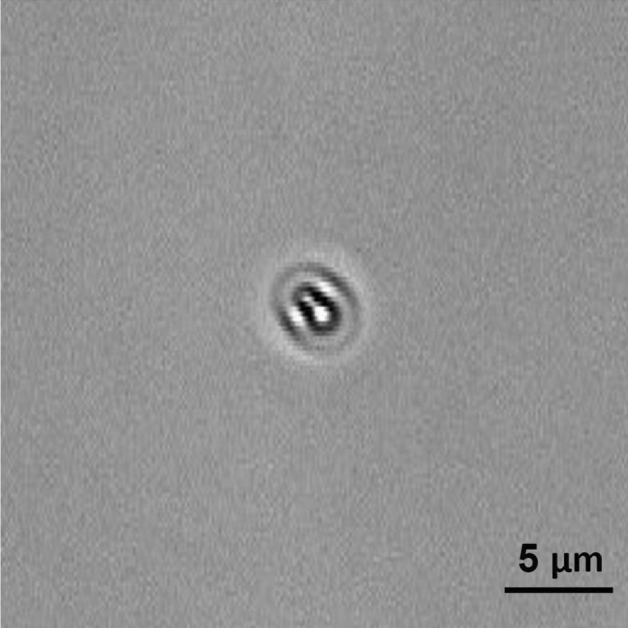
Photograph of the optical signature generated by a dead *E. coli* bacterium.

**Figure 7:**
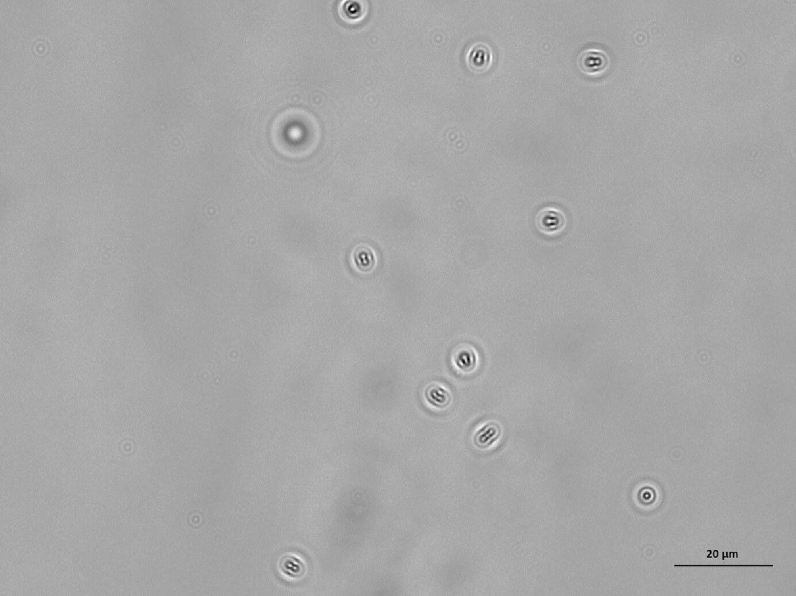
Population of dead *E. coli* bacteria dispersed in PBS and exposed to a glass surface treated with BKC for 1 h. The length of the scale bar is 20 μm.

The resultant repulsive electrostatic force generated by the interaction between the bacteria and the surface contrasts with the initial attachment and compels the bacteria to randomly diffuse above the surface, exhibiting Brownian motion (Fig. 4a). However, because of the other forces involved, such as Van der Waals (attractive) forces and gravitational forces, bacteria can overcome the electrostatic repulsion and attach to the surface. Depending on the surface area of the membrane attached to the surface and the resulting dynamics of the bacteria, it is possible to distinguish two types of attachment: rotary attachment, when just one extremity of the bacterium adheres to the surface and the bacterium exhibits a rotatory motion around the attached pole (Fig. 4b); and lateral attachment, when the bacterium adheres to the surface for a portion of its length and the bacterium exhibits translation or sliding (Fig. 4c). The same interaction dynamics was observed in bacteria dispersed both in LB and PBS (Figs. S1–S4 of the online available dataset). These types of attachment have been recently investigated experimentally using brightfield microscopy by Agladze et al., in an effort to characterise the reversible and irreversible attachment of *E. coli* bacteria. The authors concluded that lateral attachment is irreversible and that bacteria experiencing this specific adhesion do not exhibit any movement^24^. However, our results demonstrate that the initial attachment is reversible even when it is lateral. Depending on the forces acting on the bacteria, the type of attachment can change from rotary to lateral and vice versa (Figs. S5–S10 of the online available dataset). Hence, because of the variety of forces and interactions involved, the distinction between irreversible and reversible attachment should be made not on the basis of the surface area adhered but by considering the timescale of the interaction^25^. In this study, it was observed that even bacteria laterally attached to the surface were not stationary and were not simply adhered statically to the surface but exhibited a sliding movement along the surface. At this stage, bacteria are reported to start secreting extracellular polymeric substances (EPS)^6^. The EPS layer, along with the attractive Van der Waals forces and membrane pili, strengthened the adhesion and expand the surface around a bacterium which is influenced by its presence, and act as a nutrient trap facilitating the attachment of other bacteria. On the other hand, bacteria entering into contact with the glass surface coated with BKC just adhered statically to the surface, enabling the recognition of healthy bacteria able to start a biofilm from static and potentially dead bacteria (Fig. 4d). Figures 5a, 6 and 7 show caustic signatures for cells exposed to a surface coated with BKC and that were seen to be static implying the cells were dead and adhered to the surface, which was confirmed by fluorescence analysis using trypan blue (Fig. 5b), a dye able to penetrate cells with disrupted membranes^26^. BKC is a cationic surfactant well-known for its antimicrobial properties^27^. Once deposited, it confers a positive net charge to the surface, attracting the negative charge of bacteria and causing them to suddenly become statically adhered once delivered to the surface (Figs. S11–S13 of the online available dataset). Moreover, BKC is able to denature proteins and disrupt the cytoplasmic membrane of bacteria, causing their death^28^.

### *P. aeruginosa*: surface interaction

**Figure 8:**
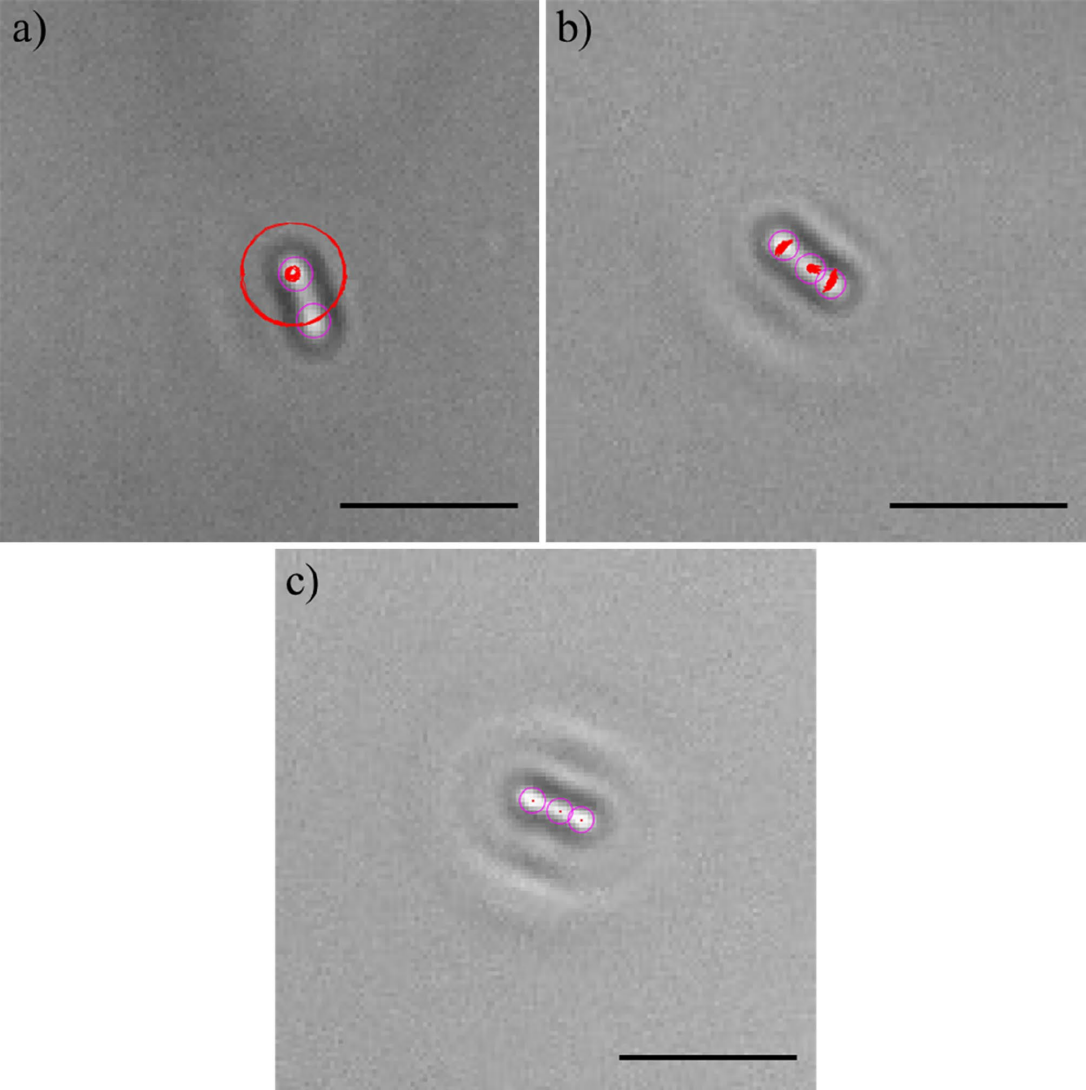
Tracks (red lines) of the sections (purple circles) of a *P. aeruginosa* bacterium: (**a**) rotary attached; (**b**) laterally attached and (**c**) static (dead). The dead bacteria were statically adhered to the surface, resulting in the absence of any noticeable tracks over time. The dynamics of the two bacteria was monitored for approximately 20 s. The length of the scale bars is 5 μm.

The analysis performed on *E. coli* bacteria was replicated on *P. aeruginosa* bacteria, to establish if the flagella-driven motility exhibited by *P. aeruginosa* bacteria influences the interaction with the surface. The results obtained suggest that once having approached the surface, *P. aeruginosa* bacteria interact with the surface exhibiting the same kind of dynamics observed for non-flagellated *E. coli* bacteria. *P. aeruginosa* attached to glass control surfaces were not static but started to rotate around the attached pole (rotary attachement, Fig. 8a) or to slide along the surface (lateral attachment, Fig. 8b). However, the presence of the flagellum seems to enhance the range of motion of bacteria attached to the surface, resulting in a more evident rotation or lateral movement along the surface (Figs. S15–S16 of the online available dataset).

As for *E. coli*, even *P. aeruginosa* bacteria exposed to glass surface coated with BKC suddenly adhered statically to the surface and did not exhibit any dynamics during the period of observation (Fig. 8c, Figs. S17–S18 of the online available dataset).

It is important to observe that random motion indicative of Brownian motion does not occur in Fig. 8 and would be expected for passive objects in a solution. Hence, it seems reasonable to consider the rotary or linear motion of bacteria adhered to a surface as indicative of life and the absence of any motion to be indicative of a dead bacteria adhered to the surface.

### Early stage biofilm formation

**Figure 9:**
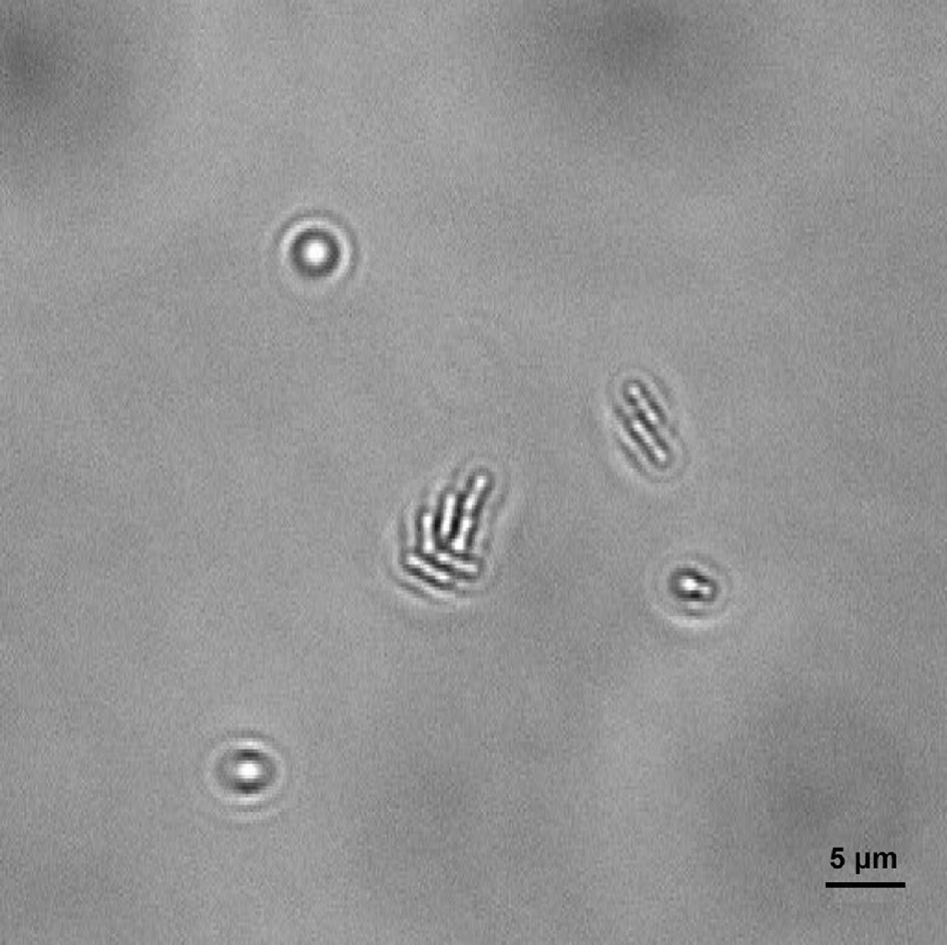
*E. coli* bacteria clustering together to start biofilms.

**Figure 10:**
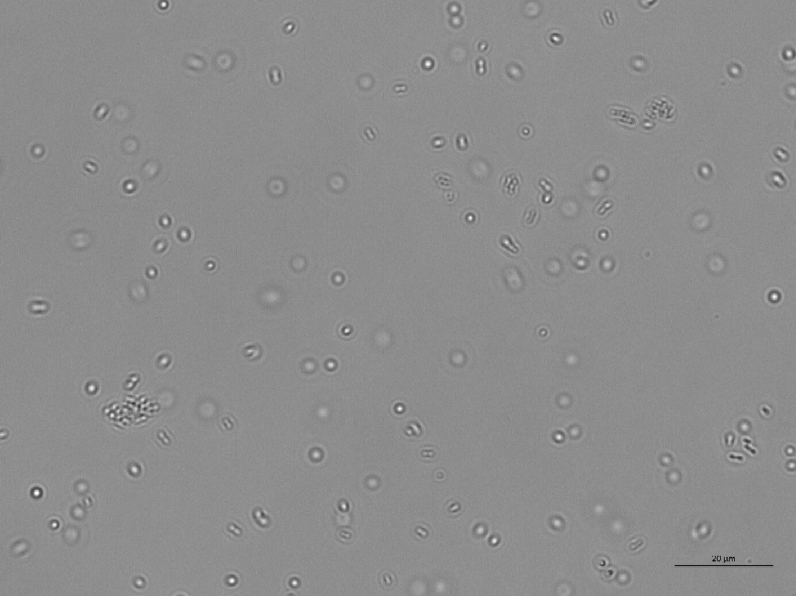
Population of *E. coli* bacteria dispersed in PBS and exposed to a glass surface for 24 h. The length of the scale bar is 20 μm.

**Figure 11:**
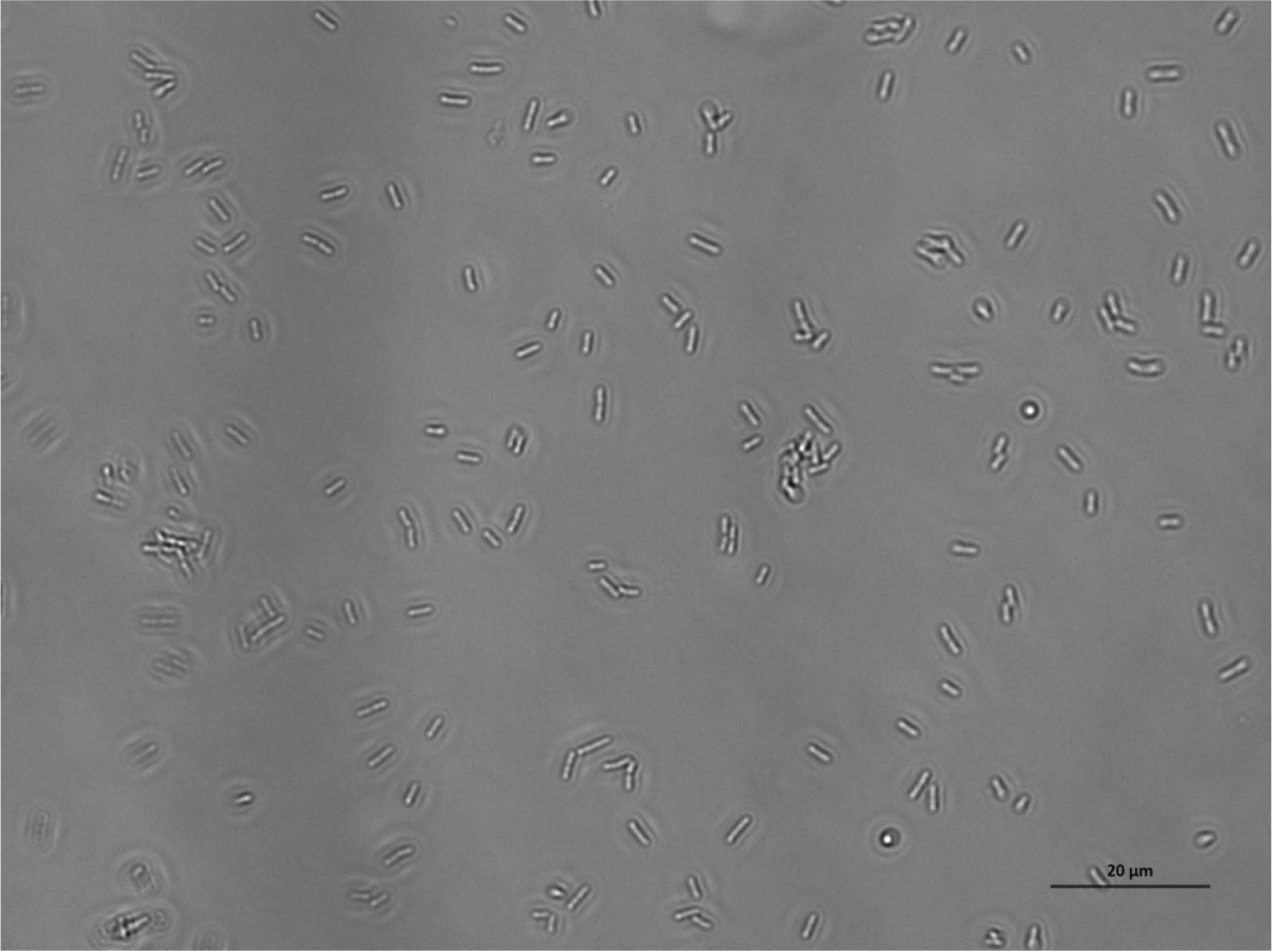
Population of *P. aeruginosa* bacteria dispersed in PBS and exposed to a glass surface for 24 h. The length of the scale bar is 20 μm.

**Figure 12:**
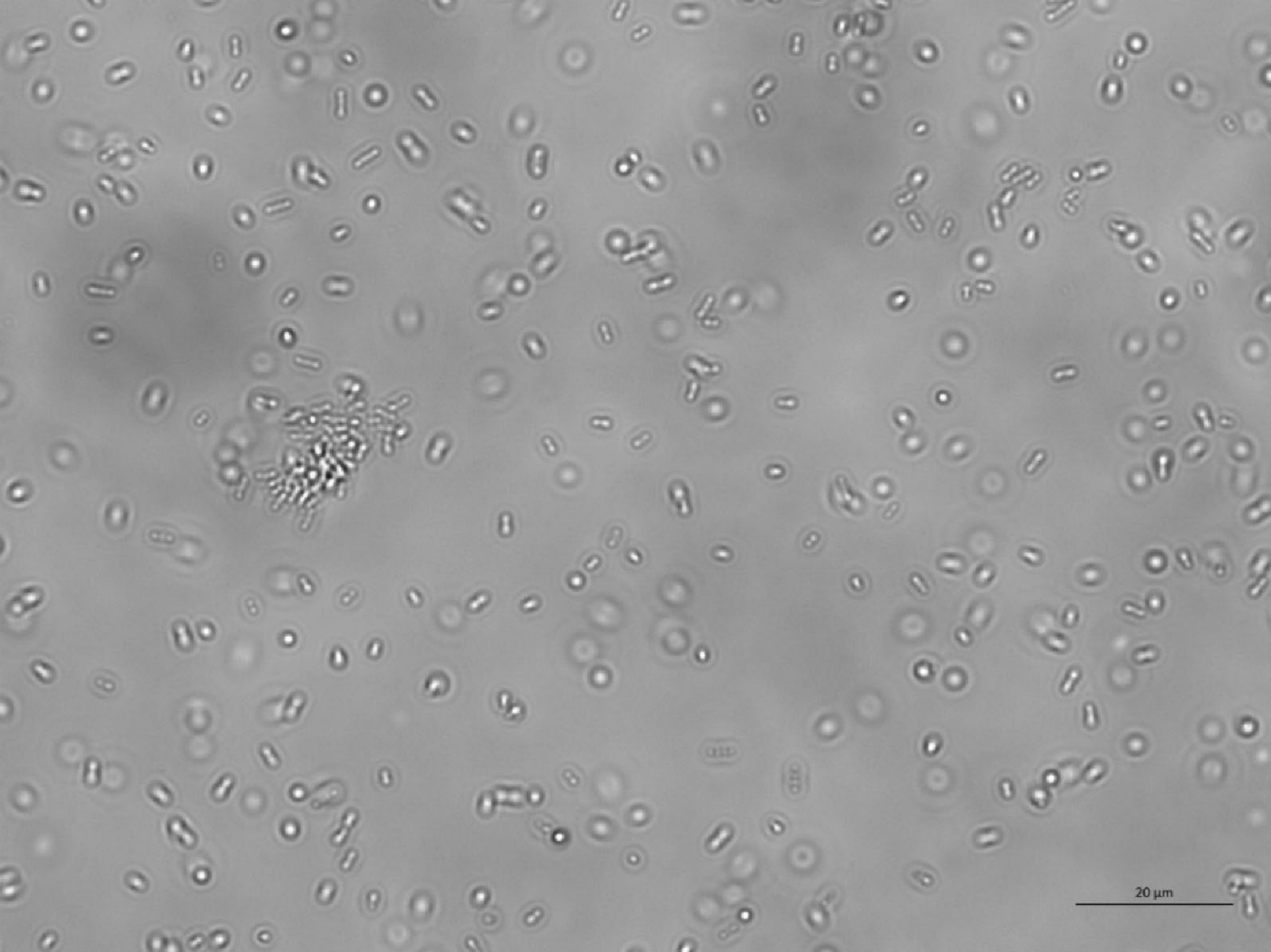
Population of *E. coli* bacteria dispersed in a solution of 10% LB in PBS and exposed to a glass surface for 24 h. The length of the scale bar is 20 μm.

**Figure 13:**
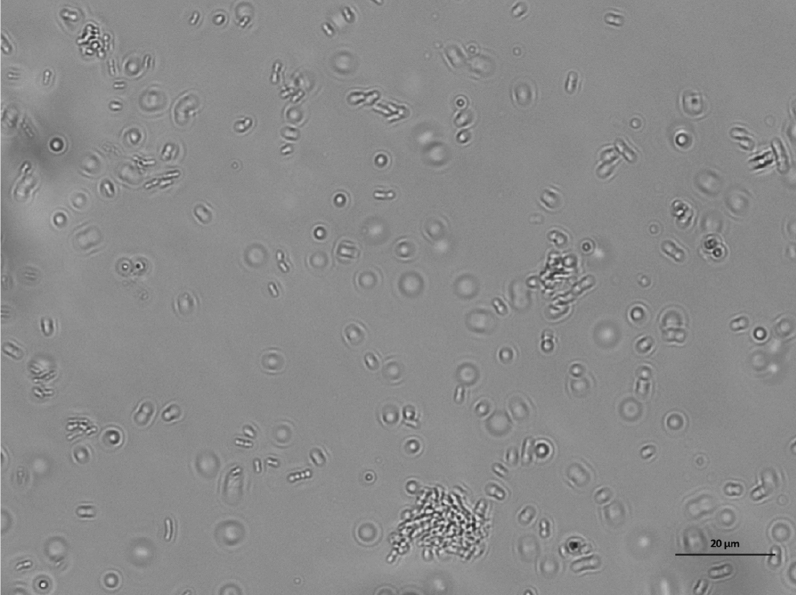
Population of *P. aeruginosa* bacteria dispersed in a solution of 10% LB in PBS and exposed to a glass surface for 24 h. The length of the scale bar is 20 μm.

**Figure 14:**
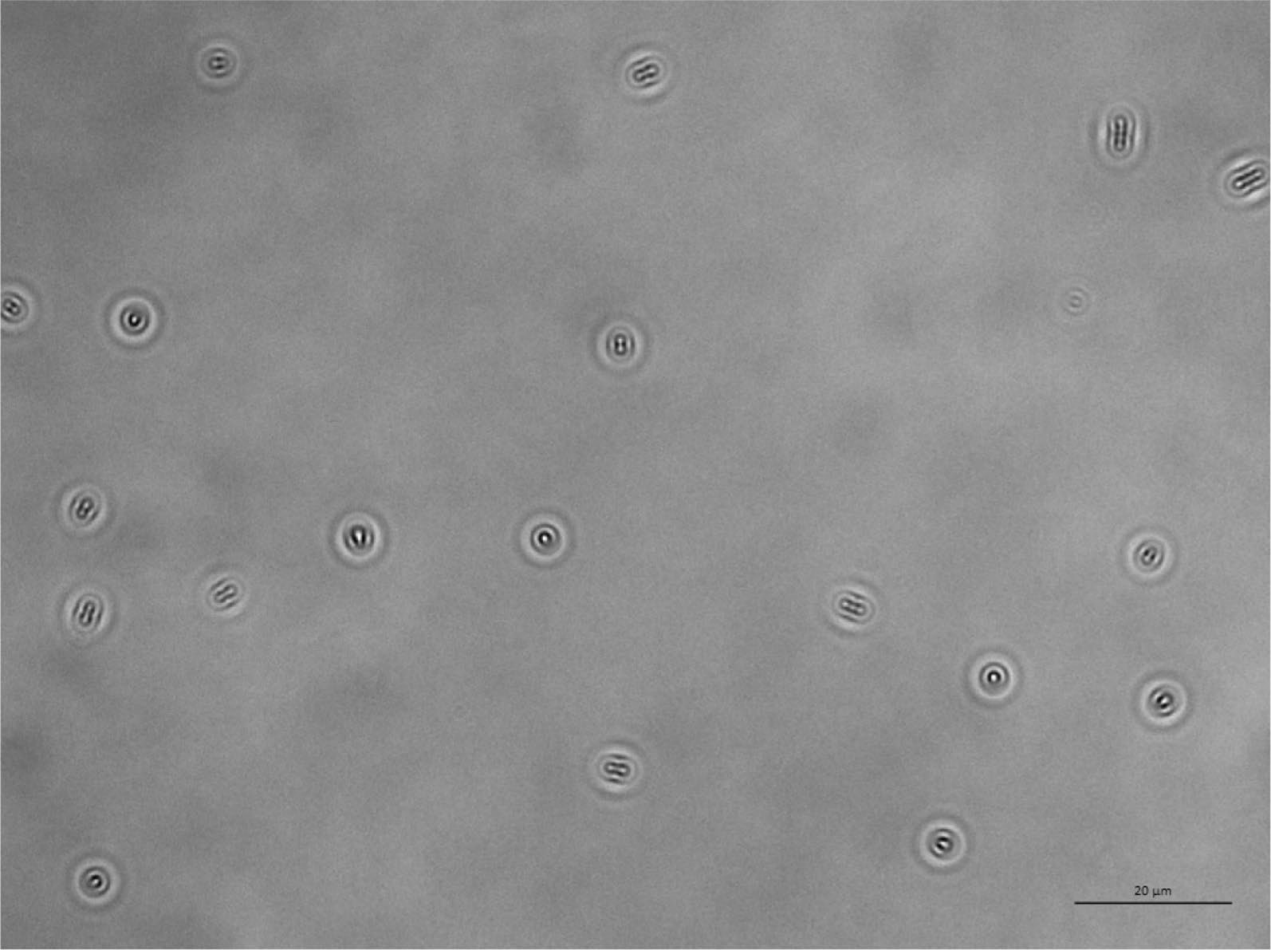
Population of dead *E. coli* bacteria dispersed in a PBS and exposed to a glass surface treated with BKC for 24 h. The length of the scale bar is 20 μm.

**Figure 15:**
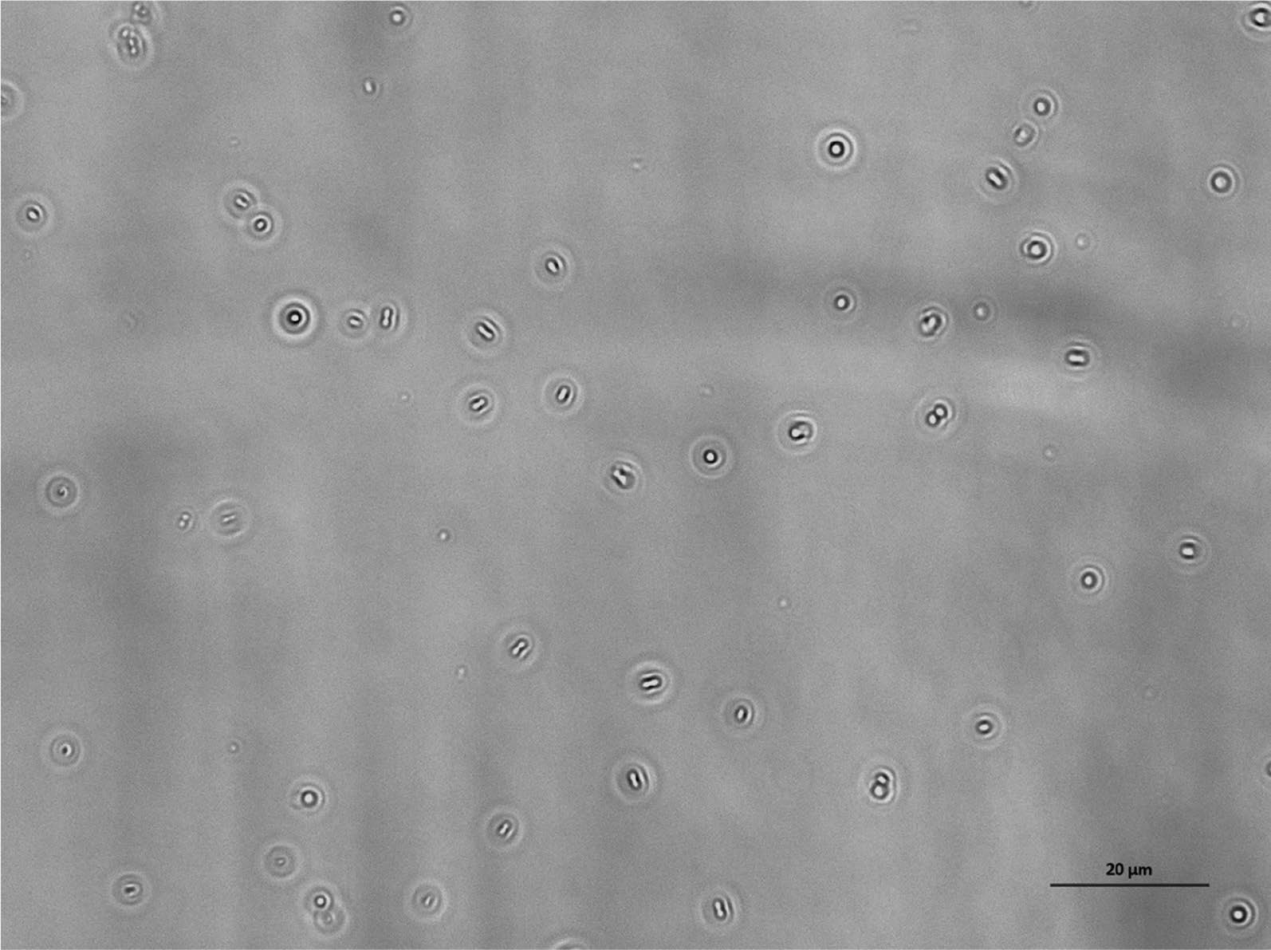
Population of dead *P. aeruginosa* bacteria dispersed in a PBS and exposed to a glass surface treated with BKC for 24 h. The length of the scale bar is 20 μm.

When exposed to untreated glass surfaces, after as little as 2 h, two or more bacteria aligned and interacted with each other, marking the initiation of the formation of a two-dimensional colony or the first stage of a biofilm^7^. Bacteria aligned in a semi-ordered way, trying to minimise the space separating them and increase the contact surface between them (Fig. 9, Figs. S19–S20 of the online available dataset). After 24 h, the concentration of bacteria and the size of the biofilm colonies increased proportionally to the amount of nutrients in solution (Figs. 10, 11, 12, 13). It is noticeable that even when a three-dimensional colony developed, it was still possible to recognise the dynamics of the system (Figs. S21–23 of the online available dataset). In fact, even at this stage of the formation of the biofilm, the bacteria were not static but would continue to secrete EPS and to dynamically interact with the surface and each other. On the other hand, the images acquired of the bacteria exposed to surfaces treated with BKC demonstrated the effectiveness of the antimicrobial compound at killing the bacteria on contact, because there was no growth in the population of bacteria and an absence of the formation of a biofilm over time (Figs. 14, 15, Fig. S24 of the online available dataset).

## Conclusion

In this paper, we have presented an experimental characterization of the mechanisms of attachment exhibited by *E. coli* and *P. aeruginosa* bacteria to control glass surfaces and glass surfaces coated with an antimicrobial agent. The results demonstrate that the viability of bacteria and their ability to start biofilms can be investigated by evaluating their dynamics and their interactions with the surface. Healthy bacteria adhered to the glass control surface exhibited detectable lateral or rotary motion. Hence, observation of these behaviours allows the early recognition of the occurrence of a potential biofilm. On the other hand, dead bacteria adhered statically to the surfaces treated with BKC, as a result of the attractive electrostatic force and of the degradation of their external membrane. Our findings not only provide new empirical insights into bacteria-surface interactions in real-time, but also have potential implications in investigations of the early formation of a biofilm or of the efficiency of an antimicrobial coating.

## Supplementary Information

Supplementary Information.

## Data Availability

The datasets generated and/or analyzed during the current study are available in the DataCat repository at: https://doi.org/10.17638/datacat.liverpool.ac.uk/1631.
