# Supplementary material for: Real-time monitoring of the dynamics and interactions of bacteria and the early-stage formation of biofilms
Source: Sci Rep. 2022 Oct 28;12:18146. doi: 10.1038/s41598-022-22669-0 (PMC9616909; doi:10.1038/s41598-022-22669-0)
Supplement: Supplementary file 1 — Supplementary Information. [file 41598_2022_22669_MOESM1_ESM.pdf]

**Supplementary Material:**

# Real-time monitoring of the dynamics and interactions of bacteria and the early-stage formation of biofilms


**Francesco Giorgi*, Judith M. Curran, and Eann A. Patterson.**

School of Engineering

University of Liverpool

Brownlow Hill

L69 3BX, Liverpool

United Kingdom

E-mail: francesco.giorgi@liverpool.ac.uk



**Keywords**: E.coli bacteria, biofilm formation, antimicrobial surface, bacteria dynamics, bacteria-surface interaction

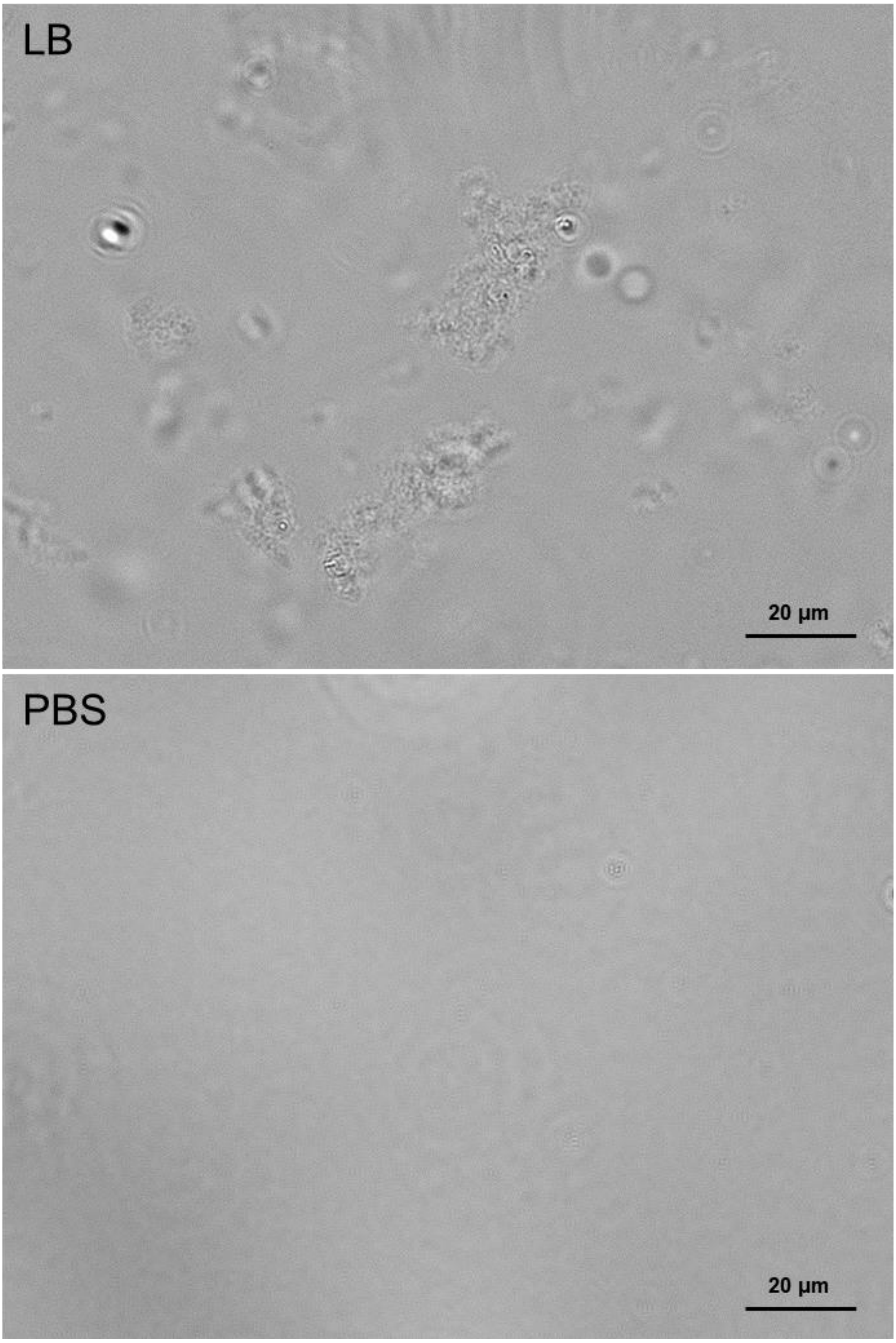


*Figure 1: Comparison between solution based on Luria-Bertani (LB) broth and Phosphate-buffered saline (PBS), imaged with an inverted optical microscope in caustics mode. The nutrients in the specific LB broth used in this study generate a visible optical signature that can potentially obscure the caustic from the bacteria in solution.*
